# Insecticidal Effects of Receptor-Interference Isolated Bioactive Peptides on Fire Ant Colonies

**DOI:** 10.3390/ijms241813978

**Published:** 2023-09-12

**Authors:** Satya Chinta, Robert Vander Meer, Erin O’Reilly, Man-Yeon Choi

**Affiliations:** 1 Center for Medical, Agricultural, and Veterinary Entomology, USDA-ARS, Gainesville, FL 32608, USA; satya.chinta@usda.gov (S.C.); erin.oreilly@usda.gov (E.O.); 2 Foresight Science and Technology, Hopkinton, MA 01748, USA; 3 Horticultural Crops Research Laboratory, USDA-ARS, Corvallis, OR 97330, USA

**Keywords:** bioactive peptides, Receptor-i, GPCR, neuropeptide, insecticide discovery, fire ant

## Abstract

Receptor-interference (Receptor-i) is a novel technology used to identify bioactive peptides as agonists or antagonists against a specific receptor, primarily targeting G-protein-coupled receptors (GPCRs). Using Receptor-i methodology, we targeted the pheromone biosynthesis activating neuropeptide receptor (PBAN-R) of the red imported fire ant (*Solenopsis invicta*). Based on previous studies, we selected four bioactive peptides cyclized with two cysteines: CVKLGSHFC, CIQQGSHFC, CERVGSHFC, and CMARYMSAC, and we conducted small-scale feeding bioassays, measuring fire ant worker mortality. All peptides reduced ant survival; however, CMARYMSAC (MARY) and CIQQGSHFC (IQQG) were the most effective and were selected for feeding trials against large, fully functional fire ant field colonies containing queen, brood, and up to 8000 workers. At the end of the experiment, day 84, synthetic peptide MARY killed over 80% of the workers and two of four queens. IQQG killed over 70% of the workers and three of four queens. The surviving two MARY queens lost an average of 21% of their starting weight. The surviving IQQG queen lost 31% of its weight. In contrast, control colony queens gained an average of 11% of their starting weight. These results provide proof-of-concept for the Receptor-i technology and will synergize applications to other agricultural and medical pests.

## 1. Introduction

Insect neuropeptides (NPs) and their receptors, primarily G protein-coupled receptors (GPCRs), are involved in essential biological processes, e.g., fat body homeostasis, feeding, digestion, excretion, circulation, reproduction, metamorphosis, and behavior [1,2,3]. Therefore, they are often proposed as biological targets for insect pest management ([4,5,6,7]. Modern biological techniques (i.e., ‘-omics’ tools) and accumulative biological data are significant in identifying these biomolecules. For example, various insect genomes, such as the i5k project (https://i5k.nal.usda.gov accessed on 21 June 2023), and RNA-Seqs, such as the sequence read archive (SRA) at NCBI (https://www.ncbi.nlm.nih.gov/sra accessed on 21 June 2023), are easily accessible for the data mining to aid in selecting target GPCRs.

Insect cell lines, such as *Spodoptera frugiperda* 9 (Sf9) cells, provide a simple and inexpensive tool to functionally express any GPCR, providing a platform for a variety of in vitro GPCR assays [8]. Ligand affinity to GPCRs can be measured by fluorescent or luminescent intensities induced by the mobilization of the second messengers, such as Ca^2+^ or cAMP molecules in the cells. This approach, coupled with an efficient technique to screen chemical libraries, is exciting and holds promise for identifying small pharmacologically active compounds for pest control [9,10,11].

Selection of target NPs and GPCRs is critical because each ligand and corresponding receptor bind extracellularly to initiate specific physiological/behavioral responses for all insect life stages. The PRXamide (-NH_2_) peptides represent a major insect NP family and are well-documented and characterized by a common C-terminal amino acid sequence, PRXamide (X = variable amino acids), which is conserved across animal groups [12,13]. These neuropeptides are grouped according to function: capability (CAPA) peptides, pyrokinin (PK) peptides, pheromone biosynthesis activating neuropeptides (PBAN), diapause hormone (DH), melanization and reddish coloration hormone (MRCH), and ecdysis-triggering hormone (ETH). The PRXamide peptides are generally encoded from three genomic groups: *capa*, *pyrokinin*, and *eth* genes.

Various bioactive PRXamide peptides or their analogues hold great potential for identification of novel active ingredients for controlling agricultural and medical arthropod pests. Insecticidal effects have been discovered against the fire ant (*Solenopsis invicta*) [14], the peach-potato aphid (*Myzus persicae*) [15,16], the Asian citrus psyllid (*Diaphorina citri*) [17], two ticks (*Ixodes scapularis* and *Rhipicephalus sanguineus*) [18,19], and the pest slug (*Deroceras reticulatum*) [20]. As agonists and/or antagonists, the short peptides identified in the above reports likely interfere with the normal operation of the target GPCR-ligand, preventing key functions in the target pest.

Ants comprise 5% of the world’s 100 worst invasive alien species, and of the 17 land invertebrates listed, 28% are ants, including fire ants in the Global Invasive Species Database (http://www.iucngisd.org/gisd/100_worst.php accessed on 21 June 2023). Like most exotic species, invasive fire ants were introduced without most of their natural enemies in their native South American range. As a consequence, fire ant populations in the United States are 5–10 times denser than in South America. In two decades, *S. invicta* has changed from an invasive pest ant in the United States to a global problem, with infestations occurring in Australia, Taiwan, mainland China, Mexico, and many Caribbean Island countries. The economic impact in the US alone is estimated to be responsible for over USD 8 billion annually for damage repair and control costs [21] with inflation adjustment. Fire ant control relies on conventional non-specific chemical insecticides. There is a need to develop targeted, biodegradable control methods.

We recently developed a new pest control paradigm referred to as “Receptor-interference” (Receptor-i) using the fire ant as a model system [14]. The Receptor-i technology can be extended to other GPCRs and other pests. The innovative methodology is comprised of the target GPCR expressed in insect cells, then screening for short peptides (7 amino acids) from phage-displayed peptide libraries using a biopanning method [14]. Selected heptapeptides showed insecticidal effects on fire ant survival after injection and feeding with synthetic peptides. The strong binding peptides are expected to be target-specific and biodegradable. In this study, we continue to explore the insecticidal effects of the peptides on fully functional (with queen and all life stages) field-collected fire ant colonies. Two heptapeptides cyclized with flanking cysteines, CMARYMSAC and CIQQGSHFC, caused significant fire ant worker mortality, queen weight loss, and a high percentage of queen death. The data support additional efforts to develop a biologically based control method for the fire ant, *Solenopsis invicta*.

## 2. Results

### 2.1. Fire Ant Worker Ingestion of Bioactive Peptides

**Figure 1:**
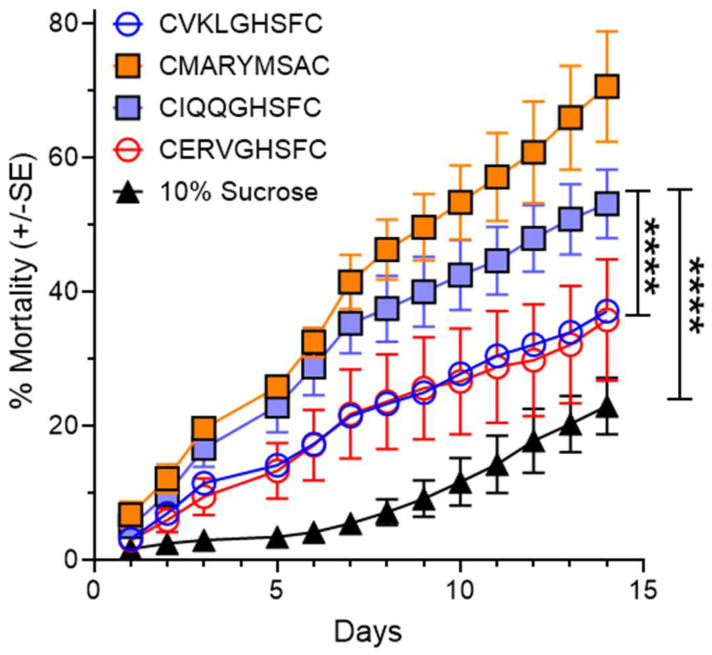
Cumulative worker mortality over 14 days is shown for 4 peptides identified through Receptor-i technology as strong binding ligands for PBAN-R. The peptides were formulated in 10% sucrose solution at 0.1% (*w*/*v*). The four peptides and sucrose control had significant linear relationships. Simple linear models with no constant were used to compare the mortality of the peptides against the sucrose control using an F-test. Each peptide slope was significantly different from the control slope. In addition, IQQG mortality over time was significantly greater than mortality associated with VKLG and ERVG. By inference, peptide MARY is significantly different from the Control and peptides, VKLG and ERVG (****, *p* < 0.0001).

Preliminary evaluations of the seven peptides using feeding acceptability and small-scale mortality studies [14] eliminated all but VKLGSHF, IQQGSHF, ERVGSHF, and MARYMSA. These peptides showed potential for further screening using a low peptide concentration (0.1% *w*/*v* in 10% sucrose). The mortalities (%) of workers treated with VKLGSHF (VKLG), IQQGSHF (IQQG), ERVGSHF (ERVG), and MARYMSA (MARY) are shown in Figure 1. Simple regression with no constant was used to test if mortality showed a linear relationship over time for the four peptides and the 10% sucrose control. All groups showed a significant linear relationship. The fitted regression model of MARY was y = 5.27x ± 0.14 (R^2^ = 0.87, F_1,38_ = 254.62, *p* < 0.0001). Peptide IQQG showed a linear relationship of y = 4.17x ± 0.13–3.5 (R^2^ = 0.82, F_1,38_ = 168.16, *p* < 0.0001). The fitted regression model of VKLG mortality was y = 2.75x ± 0.04 (R^2^ = 0.96, F_1,38_ = 940.76, *p* < 0.0001). Peptide ERVG also had a significantly linear trend of y = 2.65x ± 0.18 (R^2^ = 0.52, F_1,38_ = 40.99, *p* < 0.0001). All mortality slopes were significantly different from the control slope (y = 1.31x ± 0.09 (R^2^ = 0.63, F_1,38_ = 67.78, *p* < 0.0001).

Mortality slopes were compared to determine which peptides to move forward for further evaluations. All four peptides had a greater negative colony impact than the control (Control vs. ERVG F_1,76_ = 45.77, *p* < 0.0001, Control vs. VKLG F_1,76_ = 214.9, *p* < 0.0001, Control vs. IQQG F_1,76_ = 327.7, *p* < 0.0001, Control vs. MARY F_1,76_ = 554.7, *p* < 0.0001). However, CIQQ worker mortality was also significantly higher than ERVG and VKLG; IQQG vs. ERVG F_1,76_ = 48.61, *p* < 0.0001, IQQG vs. VKLG F_1,76_ = 110.9, *p* < 0.0001. By inference, MARY is also significantly different from ERVG and VKLG. Thus, MARY and IQQG were chosen for large-scale mortality experiments.

### 2.2. Effects of Feeding Bioactive Peptide MARY to Queen Right Colonies

**Figure 2:**
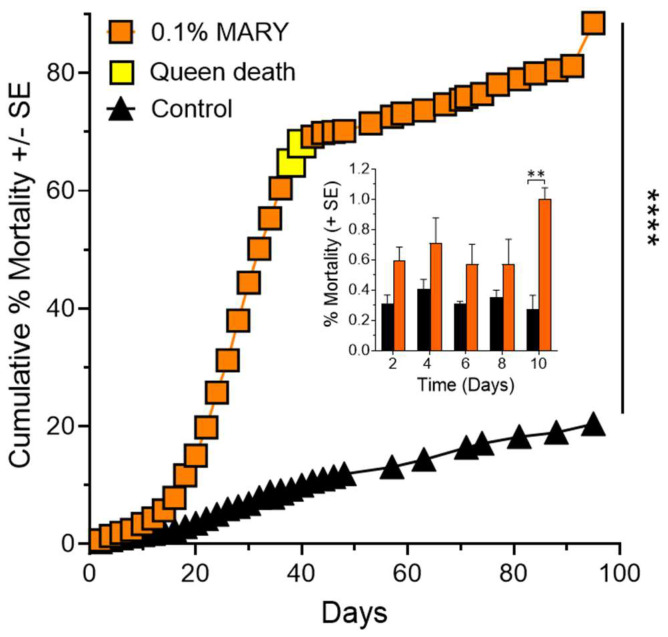
Mortality effects of Receptor-i identified peptide MARY. Colonies (*n* = 4) treated with the MARY peptide at 0.1% (*w*/*v*) in 10% sucrose solution showed significantly greater mortality when compared to the control colonies (*n* = 3) fed 10% sucrose (Kaplan–Meier survival curves), ****, *p* < 0.0001. Figure 2 Inset graph: Comparison of control and treatment colony mortality (%) during the first 10 days of the experiment. The first significant difference between control and treatment was observed at day 10 of the experiment, **, *p* < 0.01.

Queen right (fully functional) colonies (*n* = 4) fed with the MARY peptide at a 0.1% (*w*/*v*) concentration in 10% sucrose showed significantly greater mortality when compared to control colonies (*n* = 3) fed 10% sucrose (Figure 2) (Kaplan–Meier survival curves, Log-rank (Mantel–Cox) test, χ^2^ = 13,575, *p* < 0.0001).

Percent mortality was compared at each two-day period for MARY treated colonies (*n* = 4) and control colonies (*n* = 3) (Figure 2 inset). This would be an indicator of the active ingredient’s (MARY’s) latency period. Mortality (% Y-axis) per two-day period was first significantly different on day 10 (MARY $\bar{x}$ ± SE = 1.00 ± 0.07, control $\bar{x}$ ± SE = 0.28 ± 0.09, Two-way repeated measures ANOVA, F_2,8_ = 18.75, *p* < 0.01, post hoc testing Dunnett’s MCT, MΔ = 0.73 ± 0.12, Q = 6.32, df = 4.2, *p* < 0.01).

Two of four colony queens died during the experiment and the remaining two queens lost 25.52% and 15.76% of their weight by day 84. Control queens had a mean weight gain of 10.84 ± 5.04% (*n* = 3) of their starting weight.

### 2.3. Effects of Feeding Bioactive Peptide IQQG to Queen Right Colonies

**Figure 3:**
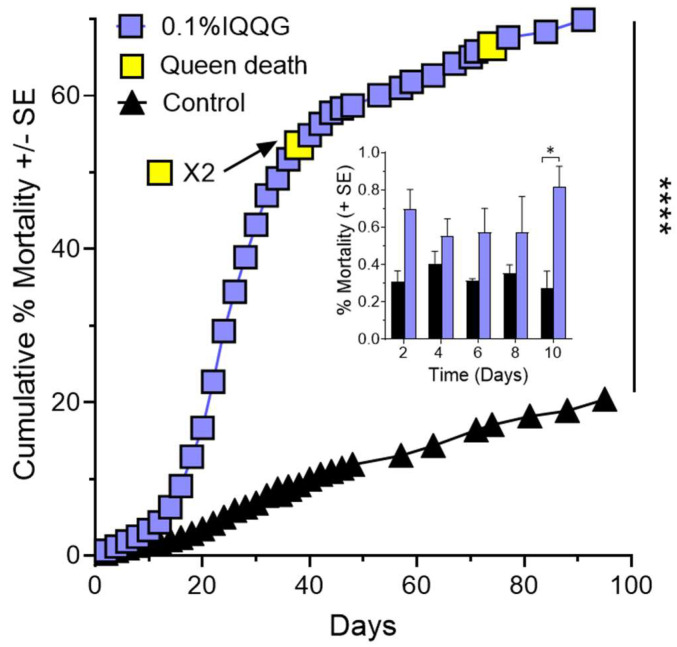
Worker and queen mortality effects of Receptor-i identified peptide IQQG on fully functional fire ant colonies (*n* = 4) treated with the IQQG at 0.1% (*w*/*v*) in 10% sucrose solution showed significantly greater mortality when compared to control colonies (*n* = 3) fed 10% sucrose (Kaplan–Meier survival curves), ****, *p* < 0.0001. Inset graph: Comparison of control and treatment mortality during the first 10 days of the experiment. The first significant difference between control and treatment was observed at day 10 of the experiment. *, *p* < 0.05.

Queen right (fully functional) fire ant colonies (*n* = 4) fed with the IQQG peptide at a 0.1% (*w*/*v*) in 10% sucrose solution, showed significantly greater mortality when compared to control colonies (*n* = 3) fed 10% sucrose solution (Figure 3) (Kaplan–Meier survival curves, Log-rank (Mantel–Cox) test, χ^2^ = 8980, *p* < 0.0001).

A two-way ANOVA was performed to analyze the effect of time and treatment on % mortality (Figure 3 inset). Dunnett’s MCT was used to compare treatments at each two-day time period. The two-way ANOVA shows that IQQG had a statistically significant effect on % worker mortality at day 10 of the experiment. IQQG 0.82 ± 0.11%, compared to the corresponding Control 0.28 ± 0.09 $\bar{x}$ ± SE. Two-way repeated measures ANOVA, F_2,8_ = 18.75, *p* < 0.01, post hoc testing Dunnett’s MCT, MΔ = 0.54 ± 0.14, Q = 3.77, df = 5, *p* < 0.05).

**Figure 4:**
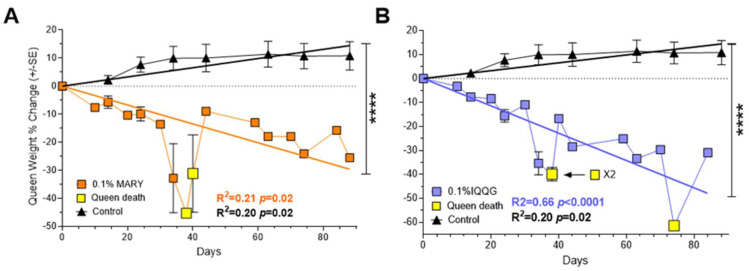
Queen weight percent change over time is shown for peptide MARY and control queens (**A**), and peptide IQQG and control queens (**B**). Significant linear regression lines and color coded R^2^ and *p* values are shown for each of the queen groups. ****, *p* < 0.0001.

Three of four colony queens died during the experiment and the remaining queen lost 30.97% of her weight by day 84. Control queens had a mean weight gain of 10.84 ± 5.04% (*n* = 3) of starting weight (see Figure 4).

### 2.4. The Effects of Bioactive Peptides MARY and IQQG on Fire Ant Queens

At the end of the experiment, peptide MARY had two surviving queens with a % weight change of −25.52% and −15.76% based on their starting weight (Figure 4A). At the last weight measurement before death (day 34), the two MARY queens had weight changes of −44.89% and −45.33%. Peptide IQQG’s surviving queen lost 30.97% of her weight by day 84, compared to the control queens with a mean weight change of +10.84 ± 5.04% (Figure 4B). The three IQQG queens that died had a weight loss of 37.1%, 42.7%, and 33.5% prior to their deaths (2 at day 38, 1 at day 74). After a colony queen died, the worker mortality counts were discontinued. Simple regression with no constant was used to test if queen weight change showed a linear relationship. MARY, IQQG, and the control queens showed a significant linear relationship between weight change and time. The fitted regression model for MARY was y = −0.34 ± 0.05x (R^2^ = 0.20, F_1,23_ = 6.04, *p* = 0.02). The fitted regression model for IQQG was y = −0.57 ± 0.05x (R^2^ = 0.66, F_1,22_ = 45.59, *p* < 0.0001). The fitted regression model for control queens was y = 0.16 ± 0.02x (R^2^ = 0.20, F_1,23_ = 5.84, *p* = 0.02). The slopes of MARY (4A) and IQQG (4B) treatments and the controls were significantly different from each other. MARY vs. Control: F_1,46_ = 93.73, *p* < 0.0001 (slopes: −0.34 vs. 0.16); IQQG vs. Control: F_1,45_ = 205.72, *p* < 0.0001 (slopes: −0.57 vs. 0.16); MARY vs. IQQG: F_1,45_ = 5.14, *p* = 0.03 (slopes: −0.34 vs. −0.57).

## 3. Discussion

### 3.1. Bioactive Peptides to the Receptor

The natural ligand PBAN, GSGEDLSYGDAYEVDEDDHPLFVPRLamide, binds to the extracellular domains of the fire ant PBAN-R, activating the receptor (= GPCR) associated with G proteins that stimulate to open specific Ca^2+^ channels embedded in the cell membrane [22,23]. None of the ten isolated short peptides reported previously [13], including the four evaluated peptides, VKLGSHF, IQQGSHF, ERVGSHF, and MARYMSA, induced the extracellular Ca^2+^ influx in the PBAN-R-transfected Sf9 cells (personal communication), indicating that the synthetic heptapeptides do not activate the fire ant PBAN-R [24]. Thus, phenotypic results using the isolated peptides are likely attributed to antagonist rather than agonist action. Interestingly, the peptide MARYMSA is unique in that it does not have --GSHF at the C-terminus like the other peptides. Peptide structural interaction with the PBAN-R will be clarified in future studies. It is anticipated that the bioactive peptides, isolated based on their strong binding to the fire ant PBAN-R, will likely exhibit specificity.

### 3.2. Hydrophobicity and Stability of Bioactive Peptides

The peptide IQQGSHF is one of the --GSHF peptide series [14]. However, MARYMSA has a unique amino acid sequence. The hydrophobicity of these two peptides is 28.6% and 57.1%, respectively. Peptides VKLGSHF and ERVGSHF have 42.9% and 28.6% hydrophobicity, respectively. It had been suggested that hydrophobic peptides are better at penetrating the midgut membrane [25]. However, our results show no correlation between hydrophobicity and fire ant mortality with these two peptides with low hydrophobicity and two peptides with high hydrophobicity (Figure 1). Therefore, peptide hydrophobicity appears to not be a critical variable for fire ant mortality. The synthetic peptides tested in this study were cyclized with two cysteine residues that increase biostability without affecting their biological activity. The structural modification of PK/PBAN with the backbone cyclic conformation was investigated and showed antagonistic activity on sex pheromone production in moths [26]. The phage display technology in our study has been widely used to isolate specific agonists, antagonists, and intracellular proteins in drug discovery [27,28]. One of the advantages of using the phage-displayed peptides is that seven amino acids produced by the bacteriophages are cyclized and exposed on their unique phage surface. The cyclic formation makes the peptide more stable without affecting biological activity [29].

### 3.3. The Ant Model Receptor-i

Ants inhabit virtually every ecosystem on Earth, exceed the combined biomass of wild birds and mammals, and equate to about 20% of total human biomass [30]. The fire ant *S. invicta* has large population densities in the southern United States and impacts many economic sectors, resulting in billions of USD spent for control and damage repair, annually [21]. Here we use the fire ant as a model system because the PBAN/PK peptides and the receptor have been well characterized in all fire ant life forms [24,31]. Our results show for the first time that the strong-binding peptides derived from Receptor-i methodology [14] have negative phenotypic effects on fully functional field collected fire ant colonies and that both colony workers and the queen are affected. This first proof of concept will synergize the use of Receptor-i technology in other applications.

### 3.4. Effect on the Fire Ant Queens

Queens mate once and have enough sperm to maintain a mature colony of 200,000+ workers for 4 to 6 years. The queens lay up to 3000 eggs daily [32]. This requires a continuous flow of food resources from the workers to the queen. Any disturbance to the flow of food resources will be reflected in the weight of the queen. For example, collecting the colonies from the field in this study temporarily broke the flow of resources to the colony queens likely resulting in weight loss that was at least partially regained after about 20 days in the laboratory feeding regime (Figure 4). However, treatment queen weights showed no indication of recovery, instead for both peptides queen % weight loss continued in a downward trajectory to the end of the experiment. Queens do not feed themselves. Workers are either impaired by the peptides and are not delivering food resources to their queen, or the queens are directly affected by the peptides fed to them by the workers, or both. It is clear from Figure 2 and Figure 3 that feeding peptides MARY or IQQG to workers led to significant worker and queen mortality or disfunction. Queen death was prefaced with large weight drops (Figure 4). Additional experiments are needed to determine the cause of queen weight loss: (1) direct effect of the peptides on the queens, (2) disruption of the worker food flow to the queens, or (3) a combination of the two. If the queen dies the colony dies, since while worker fire ants are female, they are sterile and cannot reproduce.

## 4. Materials and Methods

### 4.1. Insects

The fire ant, *Solenopsis invicta*, colonies were monogyne (single functional queen). Colonies were collected in the Gainesville, FL USA area by nest excavation or by rearing colonies from newly mated queens. All colonies were maintained as described previously [33] and were fed crickets and 10% sugar solution absorbed onto wads of tissue and maintained under standard laboratory conditions. For each set of replicates involving feeding experiments, workers from monogyne colonies were weighed/counted and placed in cups or trays whose upper inner sides were coated with Fluon^®^ (ICI Americas, Exton, PA, USA) to prevent escape. Each rearing tray contained 1 or 2 plastic Petri dish rearing cells (12 cm in diameter) depending on the number of workers and brood. Each Petri dish had a 5 mm layer of Castone dental stone (Ransom and Randolph, Maumee, OH, USA) and a hole at the center of the lid to allow easy movement of the ants in and out of the cells.

### 4.2. Peptides

Seven peptides, each with cyclized flanking cysteines (>95% purity), were synthesized by Biomatik (Wilmington, DE, USA). Their efficacy as possible fire ant (*S. invicta*) control agents was evaluated. Peptides were dissolved in sucrose solution (10%), at 0.1% working stock (*w*/*v*) solution. The peptide solution was stored at −20 °C until use in bioassays.

### 4.3. Small Scale Feeding Bioassays

This was a probing feeding bioassay where ants were continuously fed a candidate peptide and monitored daily for mortality. Each selected peptide was evaluated against workers from three monogyne fire ant colonies (in social insects the colony is the replicate). Workers, *n* = 200, from each of the three colonies were placed in plastic containers (VERSAtainer brand restaurant, 160 mm × 40 mm × 100 mm). The upper half of the inner sides of the containers was painted with Fluon to prevent escapes. Moistened cotton balls were placed in each tray to keep the ants hydrated. Test peptides (0.1% dissolved in 10% sucrose solution (*w*/*v*)) were evaluated. The control was 10% sucrose. The ants had access to the treatments and sucrose controls throughout the experiment. Worker mortality was monitored daily. After three weeks, the percent mortality was calculated and statistically compared with controls.

### 4.4. Large Scale Fully Functional Field Colony Collection and Setup

Field collected *Solenopsis invicta* colonies were excavated from populations near Gainesville, FL, USA, at sites previously established to be monogyne (single queen). Field colonies were collected by excavation into a large bucket. Workers, brood, and queen (if present) were recovered from the soil by floating out as previously described [33] and placed in a standard colony rearing tray (40 × 50 × 6 cm^3^). The inner sides of the rearing trays were coated with Fluon^®^ (ICI Americas, Exton, PA, USA) to prevent escape. Each rearing tray contained 1 to 3 plastic Petri dish rearing cells (12 cm in diameter) depending on the number of workers and brood. Each Petri dish had a 5 mm layer of Castone dental stone (Ransom and Randolph, Maumee, OH, USA) on the bottom that acted as a moisture reservoir. The lid of each Petri dish had a hole placed in the center to allow ant movement in and out of the Petri dish. After several days, field collected colonies organized themselves into the Petri dish(es) and the presence or absence of the queen could be determined. Colonies were kept only if a single physogastric queen was found. The field collected colonies were fed crickets (*Acheta domesticus*) and 10% sugar solution absorbed onto wads of tissue. These fully functional colonies contained approximately 2000–8000 worker ants and were maintained under ambient laboratory conditions (RH = 35–45%; 27–28°C; light 14: dark 10).

### 4.5. Peptide Treatments

Peptides at >95% purity were used (Biomatik) and formulated at 0.1% peptide concentrations in 10% sucrose solution. The peptide treatments were presented to the fire ant colonies in test tubes whose end was securely closed with a cotton plug. The ants then sucked the fluid from the test tubes as stimulated. The experiments were maintained continuously for 14 weeks. Worker mortality was recorded every other day. Queen weights were measured at the beginning of the experiment and every 10 days thereafter. Water (via moistened cotton ball) and crickets were provided throughout the experiment. Controls were fed water, 10% sucrose, and crickets.

### 4.6. Statistical Analysis

Statistical procedures, e.g., Kaplan–Meier Survival, and graphical representations were carried out using GraphPad Prism, version 6 (GraphPad Software Inc., San Diego, CA, USA) unless otherwise specified. Potential procedures are two-Way ANOVA; *t*-tests, non-parametric; survival analyses (Kaplan–Meier mortality curve, Log-rank, Mantel–Cox test); and linear regression. In some situations, statistical significance was determined by inspection. When multiple comparisons were required, results were inspected carrying out the analysis through specific choice of comparisons and inference. All bioassays were carried out in a manner that minimizes variance due to the environment and human activities. Details of statistical methods can be found through web search of GraphPad and Method.

## 5. Patents

There are two patents (U.S. Patent No: 9771393 B2 and U.S. Patent No: 10017358 B2) resulting from the work reported in this manuscript.

## Data Availability

Data sharing not applicable.
